# The Relationship between the Distribution of Common Carp and Their Environmental DNA in a Small Lake

**DOI:** 10.1371/journal.pone.0112611

**Published:** 2014-11-10

**Authors:** Jessica J. Eichmiller, Przemyslaw G. Bajer, Peter W. Sorensen

**Affiliations:** Department of Fisheries, Wildlife, and Conservation Biology, Minnesota Aquatic Invasive Species Research Center, University of Minnesota, Twin Cities, St. Paul, Minnesota, United States of America; CSIR- National institute of oceanography, India

## Abstract

Although environmental DNA (eDNA) has been used to infer the presence of rare aquatic species, many facets of this technique remain unresolved. In particular, the relationship between eDNA and fish distribution is not known. We examined the relationship between the distribution of fish and their eDNA (detection rate and concentration) in a lake. A quantitative PCR (qPCR) assay for a region within the cytochrome *b* gene of the common carp (*Cyprinus carpio* or ‘carp’), an ubiquitous invasive fish, was developed and used to measure eDNA in Lake Staring (MN, USA), in which both the density of carp and their distribution have been closely monitored for several years. Surface water, sub-surface water, and sediment were sampled from 22 locations in the lake, including areas frequently used by carp. In water, areas of high carp use had a higher rate of detection and concentration of eDNA, but there was no effect of fish use on sediment eDNA. The detection rate and concentration of eDNA in surface and sub-surface water were not significantly different (p≥0.5), indicating that eDNA did not accumulate in surface water. The detection rate followed the trend: high-use water > low-use water > sediment. The concentration of eDNA in sediment samples that were above the limit of detection were several orders of magnitude greater than water on a per mass basis, but a poor limit of detection led to low detection rates. The patchy distribution of eDNA in the water of our study lake suggests that the mechanisms that remove eDNA from the water column, such as decay and sedimentation, are rapid. Taken together, these results indicate that effective eDNA sampling methods should be informed by fish distribution, as eDNA concentration was shown to vary dramatically between samples taken less than 100 m apart.

## Introduction

Methods to quantify the abundance of fish populations, such as mark-recapture and electrofishing, are costly and time-consuming. In addition, fish are often difficult to capture and detect at low densities, and capture methods themselves can lead to behavioral changes of the target species [1]–[3]. Molecular methods to detect the DNA released by aquatic organisms into their environment are non-invasive, rapid, and potentially more sensitive than traditional census techniques [4]–[6]. This environmental DNA (eDNA) is released through processes such as cell sloughage, mucus excretions, and defecation [7]. Notably, eDNA is currently used to monitor the presence of invasive Bigheaded carps (often called ‘Asian carps’) (*Hypophthalmichthys* spp.) in the Chicago Area Waterway System and the Mississippi River [8]. Although initially developed as a detection tool, molecular techniques that utilize eDNA are evolving to answer more complex questions. For example, several studies have established relationships between eDNA concentration and biomass in aquatic habitats [9]–[11]. Next-generation sequencing approaches have successfully identified multiple species simultaneously [11], [12].

Despite the immense potential for eDNA technology to revolutionize monitoring programs for fish and other aquatic species, little is known about the production, fate, and distribution of eDNA in the natural environment. The distribution of eDNA is of particular importance for development of effective monitoring methods [6]. Surprisingly, Pilliod et al. [9] found that time of day, sampling location, and distance from the target organism (salamanders) had no apparent effect on eDNA concentration in small streams. In contrast, eDNA from snails was more abundant in the middle of a river channel relative to the channel margins [13]. Surface water samples are widely used for eDNA studies [8], [9], [14]. The rationale for this approach has only been confirmed in one study done in experimental ponds [15]. The possibility that eDNA concentration within a water body may be influenced by fish distribution was initially posed by Takahara et al. [10]. In a lagoon in winter, the concentration of eDNA from common carp (*Cyprinus carpio*, hereafter ‘carp’) was positively correlated with water temperature and was spatially heterogeneous. The cause of this pattern, and in particular whether it was due to the distribution of carp or higher metabolic activity of fish in warmer waters, was not examined as the distribution of carp was not measured. From the few studies that address the question of eDNA distribution in water, it is clear that it varies among habitat types, and more conclusive explanations of eDNA distribution patterns are needed. Also of interest is the distribution of eDNA in sediments, as sediments likely retain eDNA for long periods of time [16].

To determine whether fish distribution affects eDNA concentration and detection rate in lake water and sediment, we examined the distribution of carp eDNA in a small, shallow lake and compared it to known patterns of carp distribution, which had been monitored for several years. We were interested both in detection rate (percentage of samples in which eDNA levels were present above detection threshold) as well as concentration, because the former is commonly used to assess the likely distribution of invasive Bigheaded carps while the latter measure, if understood, might add more resolution and value to the technique. First, a qPCR assay specific for *C. carpio* eDNA was developed and validated in the lab. Next, since eDNA is often assumed to accumulate in surface water and sediment, surface, sub-surface, and sediment samples were taken throughout the lake. Finally, the concentration and detection rate of eDNA was compared between areas of low- and high-fish use identified from radiotelemetry data. Results of this study provide insights into optimal eDNA sampling methods for small lakes as well as information on how eDNA is distributed in aquatic systems in relation to the distribution of target organisms.

## Materials and Methods

### Quantitative PCR marker development and validation

Although two *C. carpio* qPCR assays had been developed prior to this study [10], [17], a screen against the NCBI database indicated potential non-specific amplification of non-target fish species (Table S1). Therefore, a qPCR assay was developed for the current study. Four genes were considered in the development of a novel qPCR marker specific to the common carp: (1) mitochondrial gene cytochrome *b*, (2) mitochondrial gene cytochrome *c* oxidase subunit 1, (3) mitochondrial gene control (D-loop) region, and (4) the nuclear gene recombination activating gene 1 (RAG1). Candidate primer sets were identified by NCBI Primer-BLAST using sequences under GenBank accession number X61010.1 [18] for mtDNA and EF458304.1 [19] for the RAG1 gene. Specificity was initially screened against the BLASTn database sequences for 15 fish species (Table S1). Minor groove binder (MGB) probes were manually designed using the Primer Probe Test Tool in Primer Express Software v3.0.1 (Life Technologies, Grand Island, NY). Assays with amplification efficiency outside the range of acceptable values of 90–110% or a limit of detection above 300 copies per reaction were not considered. We defined the limit of detection (LOD) as the lowest value at which three replicate reactions would successfully amplify with a quantification cycle (C*q*) value of less than 40 cycles within the linear range of the standard curve.

Candidate markers were screened for specificity for carp by testing for amplification of 15 ng of fin clip DNA from carp and 34 native and non-native fish species (Table S1). Fin clip samples for genetic marker specificity testing were extracted using the DNeasy Blood and Tissue Kit (Qiagen, Hilden, Germany) and assayed as described below.

Next, we tested markers using aqueous samples. Three 340 L flow-through tanks were set up to confirm the ability of the marker to detect carp eDNA. Prior to this experiment, all tanks were treated with 10% bleach for 30 minutes to remove all traces of DNA. The flow through rate was set at 600 mL/min, and temperature was maintained at 18°C. The first tank was stocked with 10 carp (35 g), and the second tank was stocked with 10 goldfish (*Carassius auratus*) (50 g), while the third tank was stocked with five fish of both species. These stocking levels corresponded to a biomass of 0, 438, and 875 mg/L of carp. Fish were fed once daily *ad libitum* a combination of flake feed (Color Tropical Marine Flake, Pentair Aquatic Eco-systems, Inc., Apopka, FL) and 2.5 mm pellet feed (Oncor Fry, Skretting USA, Tooele, UT) that did not contain target genetic markers. After 6 days, 4 1 L water samples were collected from each tank, immediately stored at 4°C, and filtered within 4 h. Molecular analyses followed protocols described below. This study was carried out in strict accordance with the recommendations in the Guide for the Care and Use of Laboratory Animals of the National Institutes of Health. The protocol for care and holding of laboratory fish was approved by the University of Minnesota’s Institutional Animal Care and Use Committee (IACUC) (Protocol: 1407-31659A). No anesthesia or euthanasia was required as part of this study.

### Study site

The study site was Lake Staring, a small freshwater lake located in the Upper Mississippi River Basin (44°50’14” N, −93°27’18” W). Lake Staring is a small, shallow lake that experiences frequent mixing due to wind and is typical of high carp density lakes in this region [20]. The surface area of the lake is 65.7 ha, consisting mostly of littoral zone with a depth of less than 2 m. The maximum depth is 4.8 m, and the lake bed is composed of fine sediment. Due to high carp density, the lake lacks aquatic vegetation except for white water lily (*Nymphaea odorata*), which covers less than 10% of the lake area.

Carp population abundance in Lake Staring was estimated in 2011 using a mark-recapture analysis [20]. This analysis showed that the lake was inhabited by approximately 26,000 carp, 95% CI [21,000, 31,000], or approximately 400 carp/ha. The mean body length of carp was 444 mm, indicating that the population was primarily composed of adults [20]. Approximately 14,000 fish were removed from the lake in the winters of 2012 and 2013, and a new population estimate was generated in the fall of 2013 by conducting a mark-recapture analysis. For the mark-recapture analysis, 46 carp were marked with individually numbered tags in November 2013, of which 22 were recaptured during the following four months among 5,457 carp that were captured and examined for marks. Using these data we estimated that Lake Staring was inhabited by 11,153 carp, 95% CI [7,972, 14,334] in the fall of 2013. The biomass decreased only slightly, from 490 kg/ha in 2011 to 397 kg/ha in 2013 because the mean body length of carp increased to 559 mm over this time frame. The biomass at the time of this study was approximately 20 mg/L, assuming an average lake depth of 2 m.

Since 2011, the distribution of carp has been regularly assessed by locating 10–20 carp equipped with internal radio tags (F 1850, Advanced Telemetry Systems, Isanti, MN). Carp location was determined by identification of signal directionality (bearing) with a hand-held antenna and a compass while positioned within 200 m of the radiotagged fish. Two bearings were measured for each fish, each from a different location, and their intersection calculated (LOAS, Ecological Software Solutions, CA) to estimate fish location. Mean measurement error (30 m) was estimated using dummy tags.

Using previously determined carp locations, the pattern of carp habitat usage was examined for the warm season (June–October) when carp maintain stable summertime distributions [21]. A total of 12 radiotelemetry surveys within the 2011–2013 time frame were conducted. Individual locations (N = 135) were pulled across fish and years. Areas of high carp use were estimated by calculating kernel density (search radius  = 35 m, approximately one SE of carp location estimate; output cell size  = 4.2 m) using spatial analyst in ArcMap (10.0, Esri, Redlands, CA).

**Figure 1:**
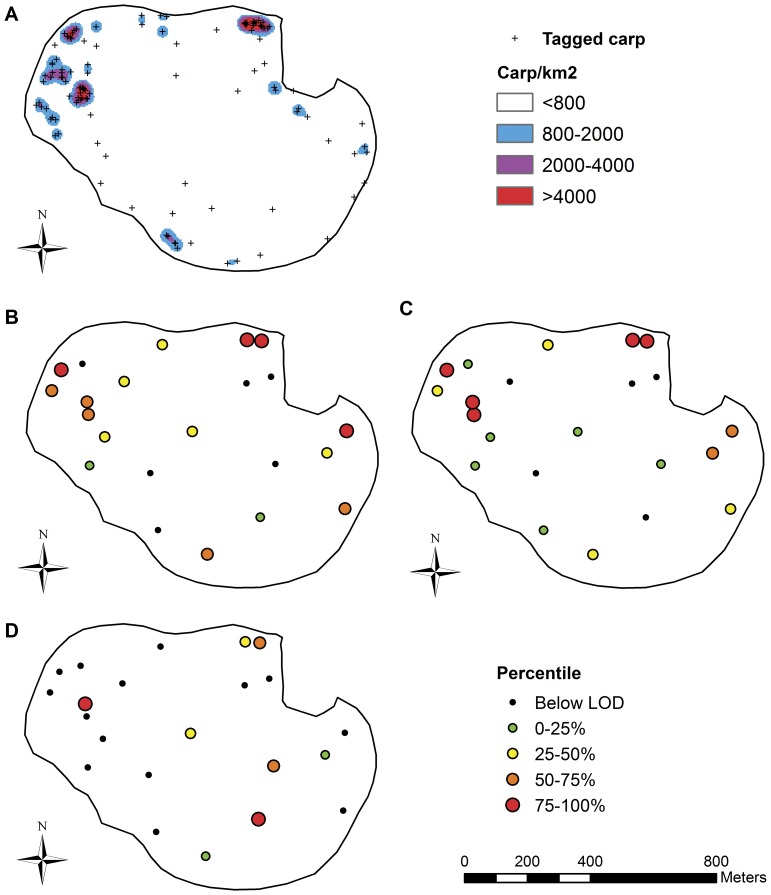
Carp use and distribution of eDNA in Lake Staring. Panel A shows locations of radiotagged carp and high- and low-use areas. Density categories represent the average number of locations of radiotagged carp/km^2^. The high- and low-use area cut-off value of 800 radiotagged carp/km^2^ corresponded to approximately 1,248 carp/ha. Panels B–D show the pattern of eDNA detection and concentration in surface water (B), sub-surface water (C), and sediment (D). All figures have the same scale. The symbol legend in the upper right refers to panel A, whereas lower right refers to panels B–D.

Carp showed well-defined areas of habitat usage in Lake Staring (Figure 1A). A density of 800 radiotagged carp/km^2^ was used as the cut-off between high- and low-use areas. As such, areas with lower values were considered to be low-use areas, whereas areas with higher densities were classified as high-use areas. The value of 800 radiotagged carp/km^2^ corresponded to approximately 1,248 carp/ha. This cut-off value was chosen because lower densities were associated with relatively isolated radiotagged carp observations. The high-use areas were located within or near the patches of lilies, which the carp most likely use for cover as the lake lacks other physical structure. In addition, all high-use areas were within 200 m of the shoreline and less than 2 m deep.

### Field sampling

Field sampling took place on 8 October 2013. Average wind speed was 20 km/h from the S [22], while air and water temperatures were 17.8°C and 19.8°C, respectively. Water and sediment samples were collected from 24 locations within the lake. Samples were taken at 18 points at 4 to 5 locations along three N to S transects of the lake and at one location at the E and W ends. Six additional sampling points were added within three patches (two samples per patch) of *N. odorata* where we knew carp were generally found. At each sampling site, surface water, sub-surface (0.5 m depth) water, and sediment were sampled. A surface water sample was also taken in both the inflow and outflow of the lake.

Water samples were collected in 1-L HDPE bottles (Nalgene, Rochester, NY) that had been previously soaked in 10% bleach for at least 30 min to remove all traces of DNA. Bottles were subsequently rinsed with distilled water to remove residual bleach. Surface water samples were taken by partially submerging a sample bottle to collect water from the top few cm. Sub-surface samples were taken using a stainless steel Van Dorn sampler (Wildlife Supply Company, Yulee, FL). Sediment samples were collected using a stainless steel Petite Ponar grab sampler (Wildlife Supply Company, Yulee, FL). Sediment was transferred to a sterile Whirl-Pak bag (Nasco, Fort Atkinson, WI) using a sterile polystyrene spatula (Bel-Art, Wayne, NJ). Once collected, samples were immediately placed on ice. Water and sediment samples were stored at 4°C and were filtered within 24 h. No specific permissions were required for access to the study site or collection of samples as part of this study. No animals were collected as part of this study.

### Molecular Analyses

Water samples were filtered through Whatman 934-AH 1.5 µm glass microfiber filters (GE Whatman, Fairfield, CT) using a polyphenylsulfone filter funnel (Pall Corporation, Port Washington, NY). Filter funnels and forceps were soaked in 10% bleach and rinsed in distilled water prior to use and between samples. For tank samples, 1 L of water was filtered per sample. For field samples, only 200 mL could be filtered per sample due to clogging from the high amount of suspended solids. Filters were stored at −80°C until DNA extraction.

Sediment samples were homogenized, and a 0.1 g subsample was stored at −80°C for DNA extraction. Preliminary experiments showed that extraction of greater than 0.1 g of sediment lead to reduction in eDNA yield and inhibition of qPCR, regardless of post-extraction inhibitor removal protocols or inclusion of PCR adjuvants. For moisture content analysis, a 10 g subsample of sediment was weighed and then dried at 100°C for 24 h.

DNA was extracted using the QIAamp DNA Stool Mini Kit (Qiagen, Hilden, Germany) using the human DNA analysis protocol. Frozen filters were sliced into 1 mm×5 mm fragments with a sterile razor blade and then transferred to extraction tubes. For sediment samples, extraction buffer was directly added to frozen sediment. Before extraction, 50 ng of UltraPure Salmon Sperm Solution (Life Technologies, Grand Island, NY) was added to adjust for extraction efficiency of DNA as previously described [23]. DNA was eluted in a final volume of 50 µL. To further remove potential inhibitors, all DNA extracts were processed with the Wizard Genomic DNA Purification Kit (Promega, Madison, WI).

A multiplex qPCR assay was designed to amplify both the CarpCyt*b* and the extraction control targets, and the oligonucleotide concentrations were optimized for this study (Table 1). CarpCyt*b* standard was created by cloning PCR product amplified from carp fin clip DNA for the CarpCyt*b* genetic marker (Table 1) using the StrataClone PCR kit (Stratagene, Santa Clara, CA). Purified plasmid DNA was quantified by using a QuantiFluor-ST Fluorometer (Promega, Madison, WI). For the extraction control, standards were created by diluting UltraPure Salmon Sperm Solution (Life Technologies, Grand Island, NY). CarpCyt*b* and extraction control standards were combined prior to preparation of five qPCR standards, ranging from 50 to 300,000 CarpCyt*b* copies and 1.6 to 10,000 pg control DNA per 5 µL.

**Table 1:** Primers and probes used for multiplex quantitative PCR.

Assay	Target	Locus	Primer/Probe	Sequence (5’ to 3’)^a^	Conc. (nM)	Ref.
CarpCyt*b*	*Cyprinus carpio*	Cytochrome *b*	CCcytbF	CTAGCACTATTCTCCCCTAACTTAC	200	This study
			CCcytbR	ACACCTCCGAGTTTGTTTGGA	200	
			CCcytbP	(6FAM) CCCTCTAGTTACACCACC (MGBNFQ)	200	
Extraction control	*Oncorhynchus keta*	ITS^b^ region 2	SketaF2	GGTTTCCGCAGCTGGG	200	[36]
			SketaR3	CCGAGCCGTCCTGGTCTA	200	
			SketaP2	(JOE) AGTCGCAGGCGGCCACCGT (BHQ-1)	100	

*^a^*BHQ1, black hole quencher-1; 6FAM, 6-carboxyfluorescein; JOE, 6-carboxy-4',5'-dichloro-2',7'-dimethoxyfluorescein.

*^b^*ITS, internal transcribed spacer.

The assay used iTaq Universal Probes Supermix (Bio-Rad, Hercules, CA). Reactions contained 12.5 µL mastermix, 10 µg bovine serum albumin (New England Biolabs Inc., Ipswich, MA) primers and probe, and water or sediment DNA in a final reaction volume of 25 µL. The volume of water and sediment DNA added to the qPCR reaction was adjusted by testing a dilution series of a subset of 5 samples from water and sediment to confirm that inhibition was not present. For sediment samples, 2.5 µL of DNA extract was added to the reaction, and for water samples 5 µL of DNA extract was added. Reaction conditions consisted of an initial denaturation at 95°C for 3 min, followed by 40 cycles of denaturation at 95°C for 15 s and an annealing and extension step at 60°C for 1 min. Each qPCR run contained triplicate reactions of standards, non-transcript controls, and samples. Amplifications were performed using the StepOnePlus Real-Time PCR System (Life Technologies, Grand Island, NY), and C*q* values were automatically determined using the system software. Sample marker concentrations were calculated on a per-run basis. All sediment values are reported per dry g.

### Statistical analyses

Detection rate of eDNA was defined as the proportion of samples that were above the qPCR assay LOD. To analyze the effects of water sample depth (surface, sub-surface), carp usage (low-use, high-use), and matrix type (water, sediment) on eDNA detection rate, the number of detections and non-detections were statistically compared using Fisher’s exact test. Fisher’s exact test was used due to low expected values (<5) in some cells.

Concentration of eDNA in water was analyzed using a three-way Analysis of Variance (ANOVA). Main effects and 2-way interactions of carp usage (low-use, high-use), water sample depth (surface, sub-surface), and lake depth (m) were examined. Lake depth was included to determine the potential for suspended sediment to affect water column eDNA concentration, with shallower depths more likely to be affected by sediment mixing into the water column. The 3-way ANOVA was restricted to sites greater than 0.5 m and less than 2 m depth (i.e. omitting 2 shallower and 7 deeper sampling sites) because of a partial confound (high-use areas were not found at depths greater than 2 m, and no low-use areas were sampled at depths less than 0.5 m). Finally, student’s t-test was used to determine whether there was a significant difference between eDNA concentration of sites included in the ANOVA and those excluded for the low-use areas. Since only 2 sites within the high-use area were excluded, no statistical comparison was done.

For all parametric descriptive analyses and statistical tests, eDNA concentrations were log_10_ transformed to achieve normal data distribution. Values below the LOD were given a value of half the LOD prior to analysis in order to reduce skewing of data. For graphical representation of eDNA concentrations across sampling points, data above the LOD were divided into four equal percentile categories. Percentiles were determined independently for the two sample types: (1) surface and sub-surface water and (2) sediment samples. All statistical tests were conducted in JMP, Version 10 (SAS Institute Inc., Cary, NC).

## Results

### Carp genetic marker development and laboratory validation

A carp-specific genetic marker (CarpCyt*b*) was developed for a 149 bp region in the cytochrome *b* gene (Table 1). This assay had an R^2^ of over 0.99 and an average PCR efficiency of 92%. The lowest copy number that all three replicate reactions reliably and successfully amplified was determined to be the assay LOD. The assay LOD was 50 copies per reaction, which corresponded to 2.0×10^4^ copies/L for water samples and 1.0×10^5^ copies/g for sediment samples (calculated from the amount of DNA extract analyzed and the volume filtered or weight extracted). The sample LOD varied slightly for individual samples depending on extraction efficiency. Further details regarding qPCR calibration curves can be found in Table S2. The average extraction efficiency was 11% for water samples and 6% for sediment samples. The assay reliably quantified up to 3.0×10^5^ copies per reaction, the highest standard tested. No amplification of non-transcript controls was observed. The assay did not amplify DNA from a selection of 34 native and non-native fish species, including Bigheaded carps and other related Cyprinids (Table S1).

In validation tests using laboratory tanks which were stocked with combinations of carp and goldfish, no CarpCyt*b* markers were detected in the tank that contained only goldfish. In the tank with 10 carp, the concentration of markers averaged 1.3×10^7^ copies/L 95% CI [1.0×10^7^, 1.6×10^7^]. In the tank with 5 each of carp and goldfish, the concentration of markers was 4.5×10^6^ copies/L, 95% CI [3.1×10^6^, 6.7×10^6^] which was significantly lower (*p* = 0.007, Student’s t-test) than that of carp alone, indicating that the presence of more individuals yielded more eDNA. On average, individual carp contributed 3.1×10^11^ copies of CarpCyt*b* in the mixed species tank and 4.4×10^11^ copies per individual in the carp only tank.

### Detection rates of eDNA in Lake Staring

The overall detection rate of CarpCyt*b* in water samples was 75% (Table 2). The distribution of eDNA was patchy, with carp eDNA detected tens of meters from sites where eDNA was not detected (Fig. 1B, C). The detection rate was not statistically different between surface and sub-surface samples (*p* = 1.00, Fisher’s exact test). However, detection rate was significantly higher in water samples collected in high-use areas (*p* = 0.009, Fisher’s exact test), a difference of nearly 40% (Table 2).

**Table 2:** Concentration and detection rates of CarpCyt*b* in water.

	Surface			Sub-surface			Total		
Carp usage	Mean (copies/L)	Detection rate (%)	N	Mean (copies/L)	Detection rate (%)	N	Mean (copies/L)	Detection rate (%)	N
	[95% CI]			[95% CI]			[95% CI]		
Low-use	3.8×10^4^	60	15	2.7×10^4^	67	15	3.1×10^4^	63	30
	[2.2×10^4^, 6.8×10^4^]			[1.8×10^4^, 4.2×10^4^]			[2.1×10^4^, 4.5×10^4^]		
High-use	2.1×10^5^	100	7	2.6×10^5^	100	7	2.4×10^5^	100	14
	[9.8×10^4^, 3.7×10^5^]			[1.3×10^5^, 3.7×10^5^]			[1.4×10^5^, 3.8×10^5^]		
Total	6.6×10^4^	73	22	5.5×10^4^	77	22	5.7×10^4^	75	44
	[3.7×10^4^, 1.2×10^5^]			[3.1×10^4^, 9.9×10^4^]			[3.9×10^4^, 8.3×10^4^]		

The overall detection rate of CarpCyt*b* in sediment was only 36% (Table 3). Similar to the water samples, detection pattern was patchy (Fig. 1D). The detection rate of CarpCyt*b* in sediment was slightly higher in high-use areas relative to low use areas, however, the difference between these use areas was not significant (*p* = 1.00, Fisher’s exact test). The detection rate of CarpCyt*b* in low-use areas in the sediment was approximately 30% less than for the average value for water samples, however, there was no difference between the detection rate (*p* = 0.11, Fisher’s exact test). The detection rate of eDNA in sediment within high-use areas was nearly 60% less than water within high-use areas, and the difference was statistically significant (*p* = 0.005, Fisher’s exact test).

**Table 3:** Concentration and detection rates of CarpCyt*b* in sediment.

	Conc. of samples above LOD		Detection rate	
	Mean (copies/g)	N	(%)	N
Carp usage	[95% CI]			
Low-use	1.2×10^5^	5	33	15
	[7.5×10^4^, 1.8×10^5^]			
High-use	2.3×10^5^	3	43	7
	[1.1×10^5^, 4.8×10^5^]			
Total	1.5×10^5^	8	36	22
	[1.1×10^5^, 2.1×10^5^]			

### Concentration of eDNA in Lake Staring

CarpCyt*b* concentration in water ranged from below the LOD (2.0×10^4^ copies/L) to 1.7×10^6^ copies/L (Table S3). The mean CarpCyt*b* concentration across all water samples was 5.7×10^4^ copies/L, 95% CI [3.9×10^4^, 8.3×10^4^] (Table 2). Most samples (84%) had less than 3.0×10^5^ copies/L. Only three samples had a marker concentration above 5.0×10^5^ copies/L, Surface water samples from the inflow and outflow streams had CarpCyt*b* concentrations of 6.0×10^4^ and 3.4×10^4^ copies/L, respectively.

Three-way ANOVA showed that carp use pattern had a significant (main) effect on eDNA concentration (Table 4). There was no significant effect of lake depth or sample depth on eDNA, and no 2-way interactions were significant (Table 4). For the subset of sampling locations considered in the ANOVA (0.5 to 2 m lake depth), in low-use areas the average CarpCyt*b* concentration was 3.3×10^4^ copies/L, 95% CI [1.8×10^4^, 5.9×10^4^], and in high use areas CarpCyt*b* concentration averaged 1.6×10^5^ copies/L, 95% CI [1.1×10^5^, 2.4×10^5^]. There was no significant difference in eDNA concentration of the shallow water sites included in the ANOVA and those excluded for the low carp use areas (*p* = 0.75).

**Table 4:** Results of a 3-way ANOVA for CarpCyt*b* marker in water samples.

Effects	df	F ratio	*P* value
Sample depth (surface, sub-surface)	1	0.07	0.79
Lake depth (m)	1	0.08	0.78
Carp use (low-use, high-use)	1	5.77	0.03*
Sample depth×Lake depth	1	0.18	0.67
Lake depth×Carp use	1	0.62	0.44
Sample depth×Carp use	1	0.26	0.62
Error	19		

For sediment samples, the concentration of eDNA ranged from below the LOD (1.0×10^5^ copies/g) to 5.4×10^5^ copies/g (Table S3). For sediment samples above the LOD, the concentration of CarpCyt*b* was slightly higher in high-use areas (Table 3), but the difference was not significant (*p* = 0.3, Student’s t-test). On a per mass basis, the lowest measureable sediment concentration of CarpCyt*b* was nearly two orders of magnitude greater than the water sample with the highest concentration of CarpCyt*b*.

## Discussion

This study found that both the detection rate and concentration of carp eDNA strongly correlated with the distribution of carp in lake water. In water, the concentration of the carp genetic marker CarpCyt*b* was over 7 times greater in high-use areas as opposed to low-use areas, and detection rate rose from 63% to 100%. The detection rate and concentration of eDNA did not differ between surface and sub-surface water samples. Detection rate was comparably low in sediment, at 36%. The distribution of eDNA is fundamentally important in the design of eDNA sampling schemes and accurate interpretation of eDNA data [6]. Thus, we have shown that the distribution of a target organism must be carefully considered in the design of eDNA sampling schemes and accurate interpretation of eDNA data. Specifically, eDNA is patchily distributed in the environment, and the probability of detecting a target organism may drastically decline tens of meters from areas that are frequently inhabited. As part of this study, a highly-specific qPCR assay for common carp, an invasive and broadly distributed fish, was developed and validated.

The patchiness of eDNA distribution within water and sediment samples taken from Lake Staring was unexpected, given that the lake is small, shallow, and has a high biomass of carp. However, Pilliod et al. [9] also observed high variation in amphibian eDNA concentration of replicate water samples in small freshwater streams. The authors hypothesized that variation was due to downstream pulses of eDNA due to activity of the target organism or variation in the cell type or form (free or cellular) of eDNA. An alternative, and perhaps complimentary explanation, is that mechanisms of eDNA removal from the water column, such as sedimentation and decay, are rapid. Lake Staring is a eutrophic lake, with high productivity and turbid, nutrient rich water. Carp eDNA is primarily contained in the particle size fraction ranging from 1.0–10 µm [24]. Particulate eDNA is continuously settling into lake sediments, but suspended particles are also hot spots of microbial degradation in aquatic systems [25]. Although decay of eDNA was not measured in this study, microcosm studies suggest that eDNA decays rapidly in the environment, and eDNA degrades nearly 90% within several days in water [12], [26], [27], [28]. Taken together, it is likely that rapid removal of eDNA from the water column through processes of decay and sedimentation prevented its accumulation and diffusion from release points in Lake Staring, leading to significantly higher concentrations of eDNA in areas where carp were present.

Regardless of the cause of eDNA’s patchy distribution, it has implications for the optimal sampling of eDNA. In low-use areas, the detection rate of eDNA was 40% lower in low-use areas as opposed to high-use areas. Distances between high-use and low-use sampling sites were tens or hundreds of meters apart, and that small distance, in some instances, affected whether eDNA was detected or not. Differences on a fine spatial scale have been observed in streams [9], [29], experimental ponds [15], and lakes [10]. Therefore, we conclude that eDNA sampling should be conducted on small spatial scales. Only after extensive testing and consideration should sampling intervals of greater distances be used.

To the authors’ knowledge, only one other study has attempted to correlate fish eDNA distribution within a lentic system. A positive relationship between carp eDNA concentration and temperature was noted by Takahara et al. [10] within a Japanese lagoon using a different marker on a slightly coarser scale without explicit information of carp distribution. The authors posited that the distribution corresponded to temperature because carp prefer warmer water; however, the data were not compared to the actual distribution of carp, as in the present study. Therefore other factors, such as higher metabolic activity of fish within warmer waters, could not be ruled out. Nevertheless, there are concordances with patterns observed between these studies. For example, excluding the lagoon channel, areas with higher eDNA appeared to have been located near the shoreline [10]. Similarly, hot spots of eDNA near shore was observed within Lake Staring, due to carp aggregation.

The overall detection rate of carp eDNA in Lake Staring water was 77%, similar to other studies conducted in areas of high fish abundance. For example, the eDNA of Bigheaded carps in the surface waters of a reach of the Mississippi River with a high target population was detected in 64% of samples [30]. Similarly, a 90% of samples were positive for common carp eDNA in a lagoon used for breeding purposes by Takahara et al. [10].

Despite the assumption of eDNA accumulation in surface waters, water depth did not affect concentration or detection rate of carp eDNA in the study lake. Therefore, both surface and sub-surface samples were equally effective for eDNA sampling in the present study. Although most eDNA samples are taken from the water’s surface [11], [14], [26], [31], only one study, done in experimental ponds, has confirmed that eDNA is most frequently detected in surface waters [15]. The convention of surface water sampling may be a holdover from early methods, wherein eDNA was used to detect floating feces of large marine mammals [32]. Our results indicate that the level of eDNA accumulation in surface water may differ among species, and accumulation likely depends on the relative proportion of eDNA sources, buoyancy of fecal material, and site-specific factors.

Although eDNA is hypothesized to accumulate in sediment, the detection rate was unexpectedly low, at 36%. This is likely due to the high LOD of the CarpCyt*b* marker in sediment. The LOD in sediment was 100,000 copies/g, nearly 4 orders of magnitude higher than for water samples on a per g basis. The high LOD is likely partly due to the limited amount of sediment that could be extracted. DNA extracts prepared with more than 0.1 g of sediment were observed to inhibit the qPCR reaction; therefore, greater amounts of sediment were not capable of being processed. Although the extraction efficiency of sediment was within the range previously observed for commercial DNA extraction kits [33], it was approximately half that of water samples. We do not know the cause of this discrepancy, but we hypothesize that sample chemistry can differentially affect extraction efficiency. Due to the difficulty of DNA recovery from sediment and the potential for qPCR inhibition, water sampling was more efficient for detection of carp in the study lake.

Regardless of the low detection rate of eDNA in sediment, its importance as a reservoir of carp eDNA cannot be disregarded. The concentration of CarpCyt*b* was high in sediment locations where eDNA was detected, but as the majority of the samples (63%) were below the LOD, we were unable to reliably calculate a mean sediment eDNA concentration. The accumulation of eDNA in sediment has been suspected based on the high concentration of microbial DNA in sediment [34], [35], but measurements of fish eDNA in sediment have not been previously published. Hot spots of eDNA in sediment did not correlate with carp use. Therefore, there is a need for future studies to measure factors that may control eDNA distribution in sediment, such as deposition, resuspension, and degradation rates.

## Conclusions

Sampling design has been previously identified as one of the four critical aspects that must be optimized in a DNA-based monitoring program [6]. The present study showed that common carp distribution led to spatial patterns in both eDNA concentration and detection rate in a small, shallow lake. Our results show that while eDNA is relatively evenly distributed in the water column, eDNA is patchily distributed horizontally. The large variation of eDNA on a small spatial scale, of tens to hundreds of meters, indicates that sampling for aquatic species using eDNA should use a similarly fine scale, at least for initial surveys. The results of this study also indirectly suggest that mechanisms of eDNA removal from the water column are rapid and may partially control eDNA distribution. Although the observations of the current study may not be universally applicable to all species and habitats, our results indicate that eDNA sampling schemes should be critically evaluated for the specific organism and the type of aquatic environment they inhabit. Future research is needed to examine the role of decay, sediment re-suspension, and eDNA release on eDNA distribution in aquatic habitats.

## Supporting Information

Table S1 List of fish species tested for marker specificity.
(DOCX)

Table S2 Quantitative PCR calibration data.
(DOCX)

Table S3 Coordinates, lake depth, fish use, and eDNA concentration at sampling sites.
(DOCX)
