# Supplementary material for: The Relationship between the Distribution of Common Carp and Their Environmental DNA in a Small Lake
Source: PLoS One. 2014 Nov 10;9(11):e112611. doi: 10.1371/journal.pone.0112611 (PMC4226586; doi:10.1371/journal.pone.0112611)
Supplement: Table S1 — List of fish species tested for marker specificity. (DOCX) [file pone.0112611.s001.docx]

| **Scientific name** | **Common name** | **Potential for non-specific amplification of previously published *C. carpio* markers***^a^* | | **BLASTn screening** | **CarpCyt*b* amplification** |
| --- | --- | --- | --- | --- | --- |
|  |  | Takahara et al., 2012*^b^* | Mahon et al., 2013*^c^* |  |  |
| *Ambloplites rupestris* | Rock bass |  |  |  |  |
| *Ameiurus melas* | Black bullhead |  |  | X |  |
| *Ameiurus natalis* | Yellow bullhead |  |  |  |  |
| *Amia calva* | Bowfin |  |  |  |  |
| *Aplodinotus grunniens* | Freshwater drum |  |  | X |  |
| *Carassius auratus* | Goldfish | X | X | X |  |
| *Carpiodes cyprinus* | Quillback |  |  |  |  |
| *Catostomus commersonii* | White sucker |  |  |  |  |
| *Ctenopharyngodon idella* | Grass carp |  |  |  |  |
| *Culaea inconstans* | Brook stickleback |  |  |  |  |
| *Cyprinella spiloptera* | Spotfin shiner |  | X |  |  |
| *Cyprinus carpio* | Common carp |  |  |  | X |
| *Dorosoma cepedianum* | Gizzard shad |  |  |  |  |
| *Esox lucius* | Northern pike |  |  | X |  |
| *Esox masquinongy* | Muskellunge |  |  | X |  |
| *Etheostoma nigrum* | Johnny darter |  |  |  |  |
| *Hypophthalmichthys molitrix* | Silver carp |  | X | X |  |
| *Hypophthalmichthys nobilis* | Bighead carp |  |  | X |  |
| *Ictalurus punctatus* | Channel catfish | X |  | X |  |
| *Ictiobus bubalus* | Smallmouth buffalo | X | X |  |  |
| *Ictiobus cyprinellus* | Bigmouth buffalo | X | X |  |  |
| *Lepisosteus platostomus* | Shortnose gar |  |  |  |  |
| *Lepomis cyanellus* | Green sunfish |  |  | X |  |
| *Lepomis gibbosus* | Pumpkinseed | X |  | X |  |
| *Lepomis macrochirus* | Bluegill |  |  | X |  |
| *Micropterus dolomieu* | Smallmouth bass |  |  |  |  |
| *Micropterus salmoides* | Largemouth bass |  |  | X |  |
| *Moxostoma carinatum* | River redhorse | X |  |  |  |
| *Notemigonus crysoleucas* | Golden shiner |  |  |  |  |
| *Notropis volucellus* | Mimic shiner |  | X |  |  |
| *Perca flavescens* | Yellow perch |  |  | X |  |
| *Pimephales promelas* | Fathead minnow |  | X |  |  |
| *Pomoxis nigromaculatus* | Black crappie |  | X | X |  |
| *Sander vitreus* | Walleye |  |  | X |  |
| *Umbra limi* | Central mudminnow |  |  |  |  |

*^a^*Less than two mismatches within 5 bp of the 3’ end of both forward and reverse primers.

*^b^*Takahara T, Minamoto T, Yamanaka H, Doi H, Kawabata Z (2012) Estimation of fish biomass using environmental DNA. PLoS One 7: e35868.

*^c^*Mahon AR, Jerde CL, Galaska M, Bergner JL, Chadderton WL, et al. (2013) Validation of eDNA Surveillance Sensitivity for Detection of Asian Carps in Controlled and Field Experiments. PLoS One 8: e58316.
