# Supplementary material for: The Relationship between the Distribution of Common Carp and Their Environmental DNA in a Small Lake
Source: PLoS One. 2014 Nov 10;9(11):e112611. doi: 10.1371/journal.pone.0112611 (PMC4226586; doi:10.1371/journal.pone.0112611)
Supplement: Table S2 — Quantitative PCR calibration data. (DOCX) [file pone.0112611.s002.docx]

| **Assay** | **Equation***^a^* | **Range E***^b^* | **Range R^2^** | **LOD***^c^* |
| --- | --- | --- | --- | --- |
| CarpCyt*b* | *y* = –3.52*x* + 43.8 | 89 – 97 | 0.992 – 0.997 | 50 |
| Extraction control | *y* = –3.64*x* + 32.9 | 84 – 96 | 0.991 – 1.000 | 1 |

*^a^*Average calibration equation across 6 runs.

*^b^*Amplification efficiency = (10^(1/–slope)^ – 1)×100.

*^c^*Limit of detection in genetic marker copies per assay. For extraction control, LOD is in pg salmon sperm DNA.
