# Supplementary material for: The Relationship between the Distribution of Common Carp and Their Environmental DNA in a Small Lake
Source: PLoS One. 2014 Nov 10;9(11):e112611. doi: 10.1371/journal.pone.0112611 (PMC4226586; doi:10.1371/journal.pone.0112611)
Supplement: Table S3 — Coordinates, lake depth, fish use, and eDNA concentration at sampling sites. (DOCX) [file pone.0112611.s003.docx]

| **Site** | **Easting** | **Northing** | **Lake Depth (m)** | **Fish use** | **eDNA (copies/L or g)** | | |
| --- | --- | --- | --- | --- | --- | --- | --- |
|  |  |  |  |  | **Surface** | **Sub-surface** | **Sediment** |
| 1 | 464054 | 4964666 | 1.5 | Low | 162 855 | 84 272 | 82 335 |
| 2 | 464222 | 4964784 | 2.3 | Low | 51 604 | <LOD | 234 928 |
| 3 | 464270 | 4964953 | 4 | Low | <LOD | 22 710 | 176 552 |
| 4 | 464496 | 4965058 | 1 | Low | 374 785 | 121888 | <LOD |
| 5 | 463897 | 4964743 | 1.1 | Low | <LOD | 21703 | <LOD |
| 6 | 463873 | 4964924 | 3.6 | Low | <LOD | <LOD | <LOD |
| 7 | 464006 | 4965056 | 2.3 | Low | 71 770 | 23 972 | 85 075 |
| 8 | 464178 | 4965209 | 1.6 | Low | <LOD | <LOD | <LOD |
| 9 | 464256 | 4965230 | 1.2 | Low | <LOD | <LOD | <LOD |
| 10 | 463680 | 4964948 | 1.5 | Low | 26 741 | 25 438 | <LOD |
| 11 | 463728 | 4965039 | 2.6 | Low | 69 087 | 22 881 | <LOD |
| 12 | 463790 | 4965215 | 2.9 | Low | 90 951 | <LOD | <LOD |
| 13 | 463911 | 4965332 | 1 | High | 83 403 | 76 464 | <LOD |
| 14 | 464491 | 4964811 | 1.6 | Low | 101 525 | 80 089 | <LOD |
| 15 | 464434 | 4964988 | 2 | Low | 74 703 | 119 642 | 76 450 |
| 16 | 463657 | 4965271 | 1.5 | Low | <LOD | 23 507 | <LOD |
| 17 | 463590 | 4965252 | 0.9 | High | 357 558 | 226 648 | <LOD |
| 18 | 463560 | 4965186 | 0.8 | High | 99 200 | 91 533 | <LOD |
| 19 | 463676 | 4965110 | 1.1 | High | 117 578 | 310 600 | <LOD |
| 20 | 463672 | 4965150 | 1.2 | High | 172 404 | 360 571 | 539 416 |
| 21 | 464180 | 4965347 | 0.5 | High | 189 484 | 490 750 | 100 555 |
| 22 | 464226 | 4965344 | 0.5 | High | 1 684 530 | 955 076 | 224 388 |
